# Epilepsy; Its Pathology and Treatment

**Published:** 1890-11

**Authors:** 


EPILEPSY, ITS PATHOLOGY AND TREATMENT. By Hobart Amory Hare, M. D. (University of Pennsylvania), B. Sc. F. A. Davis, publisher, Philadelphia, and London, 1890.

Among the best of the *series* of medical works issued by different publishers for the instruction of the profession is *The Physicians' and Students' Ready Reference Series*, published by Mr. F. A. Davis, of Philadelphia. They are octavo volumes, excellently printed, and neatly bound. The work now under notice is No. 7 of the series.

Its author is a rising young Philadelphia physician, who, though he has been but a few years in practice, has drawn considerable attention to himself by his brilliant work as an investigator, a teacher, and an author.

This is the essay for which the author was awarded a prize of four thousand francs by the Royal Academy of Medicine, of Belgium, last December.

The essay is an exhaustive and well-written treatise upon epilepsy, conveniently divided off into paragraphs, with titles in heavy black type, agreeably to the plan in all the members of the series. In this book one can find recorded all that is worth knowing concerning epilepsy in a condensed form. It is, in fact, an encyclopædia of this strange disease, with the information concerning it well arranged for quick reference. The view of the author is that the epileptic seizure, which he very aptly terms the "nerve storm," originates in the cortical substance of the brain, and consists essentially of a sudden "explosion of nerve force." Considerable evidence is brought forward to sustain such a view.

The treatment, of course, we cannot indorse, the recommendation being chiefly for the bromides, especially the Bromide of Potassium, in immense doses. The author rather inconsistently says that no poisoning results from such quantities, yet warns his reader against the dangers of Bromism.

If the writer were but convinced of the truth of Homœopathy and of the utility of its therapeutics, and could have added an exposition of them as able and as thorough as his description of the disease, the book would have been almost perfect. W. M. J.

